# Bioprospecting for *Isoetes cangae* Endophytes with Potential to Promote Plant Growth

**DOI:** 10.1155/2023/5992113

**Published:** 2023-08-21

**Authors:** Danielle Silveira Santos, Paula Veronesi Marinho Pontes, Analy Machado de Oliveira Leite, Aline Lemos Ferreira, Mariana de Souza, Thainá dos Santos Silva Araujo, Henrique Fragoso dos Santos, Guilherme Correa de Oliveira, José Augusto Bitencourt, Allysson Buraslan Cavalcanti, Rodrigo Lemes Martins, Francisco De Assis Esteves

**Affiliations:** ^1^Federal University of Rio de Janeiro, Instituto de Biodiversidade e Sustentabilidade, Macaé 27965-045, Brazil; ^2^Fluminense Federal University, Marine Biology Department, Niterói 24001-970, Brazil; ^3^Vale Technological Institute, Belém, Pará 66055-090, Brazil; ^4^Vale S.A., Belo Horizonte, MG, Brazil

## Abstract

*Isoetes cangae* is a native plant found only in a permanent pond in Serra dos Carajás in the Amazon region. Plant-associated microbial communities are recognized to be responsible for biological processes essential for the health, growth, and even adaptation of plants to environmental stresses. In this sense, the aims of this work were to isolate, identify, and evaluate the properties of endophytic bacteria isolated from *I. cangae.* The bioprospecting of potentially growth-promoting endophytes required the following steps to be taken: isolation of endophytic colonies, molecular identification by 16S rDNA sequence analysis, and evaluation of the bacterial potential for nitrogen fixation, production of indole acetic acid and siderophores, as well as phosphate solubilization and mineralization. *Bacillus* sp., *Rhizobium* sp., *Priestia* sp., *Acinetobacter* sp., *Rossellomorea* sp., *Herbaspirillum* sp., *Heyndrickxia* sp., and *Metabacillus* sp., among other bacterial species, were identified. The isolates showed to be highly promising, evidencing the physiological importance for the plant and having the potential to promote plant growth.

## 1. Introduction

*Isoetes cangae* is an ancient plant whose evolutionary process dates from the Devonian period [1]. However, its discovery was made a few years ago by Pereira et al. [2]. The new species was named in reference to its habitat, on canga soil, in rupestrian fields. Until now, it has only been found in a permanent pond in Serra dos Carajás, Pará. This aquatic plant lives underwater and grows among the rocks in the pond, in an iron-rich oligotrophic environment. Morphologically, it is characterized by the presence of root, corm, and leaves.

Pereira et al. [2] suggest that *I. cangae* be classified as critically endangered because it was found only in a single location within a mining area, predicting a significant decrease in individuals. Indeed, in 2019, it was included in the IUCN red list as “Critically Endangered” [3]. It is, therefore, important to understand the bacteria that live in symbiosis with these plants, promoting their health, protection, and growth.

Plant-associated microbial communities are responsible for biological processes essential for the health, growth, and even adaptation of plants to environmental stresses. The benefits of this relationship are credited to compounds produced by bacteria, such as growth-regulating hormones [4], antibiotics and siderophores [5], as well as nitrogen fixation [6], and mineralization and solubilization of nutrients such as phosphorus [7].

Using bacteria to speed up the growth and promote the adaptation of plants to biotic and abiotic stresses is a widely used technology worldwide. Most times, this is the difference in the success of cultivation [8]. Amorim and Melo [9] highlight that beneficial bacteria can help in all plant cycles, allowing increase in germination rate, reproductive organ development, and productivity.

Studies on plant growth enhancement are usually carried out using rhizobacteria, but some studies point to a greater efficiency of endophytic bacteria [10]. Some of the bacteria become facultative intracellular endophytes and competitively colonize plant roots. They offer several benefits to the plant because they are in an environment that suffers less impact from the abiotic and biotic variations of the rhizosphere [11, 12].

Thus, the aims of this study were to isolate and identify endophytic bacteria with an important role in the development of *I. cangae*. The bioprospecting of endophytic bacteria with potential as growth promoters required the following steps to be taken: isolation of endophytic bacteria associated with *Isoetes cangae*, morphological description, characterization, and preservation of microorganisms and assessment of nitrogen fixation potential, production of indole acetic acid, and siderophores and phosphate mineralization.

## 2. Materials and Methods

*Isoetes cangae* is endemic of Amendoim pond, a permanent pond located in southeastern Pará State, within the limits of the Carajás National Forest-FLONA (05°52′00″-06°33′00″S and 049°53′00″-050°45′00″W). Amendoim pond has an area of 13.96 ha and a maximum depth of 7.8 m [13], with an average variation in the water column height of 2.5 m over the year. The Chico Mendes Institute of Biodiversity of the Ministry of the Environment (ICMBio/MMA; numbers 64187 and 5972) grants the permit for all collections.

### 2.1. Isolation of Endophytic Microorganisms from *I. cangae*

The bacterial isolation was carried out using 3 plants collected from 3 different portions (West, North, and East) of Amendoim pound which were processed by separating root, corm, and leaves and disinfected in 70% v/v ethanol for one minute, 2.5% v/v sodium hypochlorite solution for 3 minutes, 70% v/v ethanol for 1 minute, and two rinses in sterile distilled water [14]. Subsequently, the samples were aseptically macerated and cultured on Luria–Bertani (LB) agar (triptona, 1 g/L; yeast extract, 0.5 g; NaCl, 1 g; and agar, 20 g/L). The media were sterilized by autoclaving at 121°C for 15 min before use. The plates were incubated at room temperature for 24–48 h.

Differences in colony morphology of grown bacteria were observed: shape, elevation, margin (edges of colonies), color, surface appearance, density, consistency, and pigmentation [15]. Afterward, the cultures were tested using Gram-staining. Conservation and maintenance procedures in the short and medium term were performed to stock the grown cultures; thus, the bacterial isolates were kept frozen at −20°C in 20% glycerol, and a batch was kept at 4°C on inclined test tube Luria–Bertani (LB) agar, and mineral oil was added.

For activation of cultures at the beginning of the tests, the isolates were inoculated in LB broth (tryptone, 1 g/L; yeast extract, 0.5 g; and NaCl, 1 g), modified from Ambrosini and Passaglia [16]. The media were sterilized in an autoclave at 121°C for 15 minutes before use. The flasks were incubated at 28 ± 1°C and 100 rpm for 18 h.

### 2.2. Nitrogen Fixation Capacity

To identify nitrogen-fixing bacteria, the isolates were cultured in test tubes containing 10 mL of NFb culture medium (5 g of malic acid/0.5 g of K_2_HPO_4_/0.2 g of MgSO_4_.7H_2_O/0.1 g of NaCl/0.01 g of CaCl_2_.2H_2_O/4 mL of FeEDTA-1.64%/2 mL of bromothymol blue-0.5%/2 mL of micronutrient solution/1.75 g/L of agar; 1 L of distilled water, pH 6.8). The flasks were incubated at 28 ± 1°C for 7 days, and those that presented a typical aerotaxis film close to the medium surface were considered positive for nitrogen fixation. The test was performed in triplicate. The presence of bacterium film formation in NFb semi-solid medium as described by Kuss et al. [17]. The change in the medium's color from intense green to bluish-green or blue is an indicator that biological fixation has occurred, since it indicates the reduction of atmospheric nitrogen to ammonia.

PCR was used to detect the presence of the nifH gene in the isolates selected as nitrogen fixers, using the primers Ueda19F (5′GCIWTYTAYGGIAARGGIGG 3′) [18] and R6 (5′GCCATCATYTCICCIGA 3′) [19]. Each reaction was performed in a volume of 50 *μ*l inside a tube containing 10 l 5x PCR buffer (Promega Corporation/Madison, Wisconsin, EUA), 2 mM MgCl_2_ (Promega Corporation/Madison, Wisconsin, EUA), 200 *μ*M each of the four dNTPs (Promega Corporation/Madison, Wisconsin, EUA), 0.2 mM of each primer (IDT), and 1.5 U of Taq DNA polymerase (Promega Corporation/Madison, Wisconsin, EUA). The program used for amplification followed the steps: 95°C for 5 minutes, followed by 35 cycles of 94°C for 30 seconds, 52°C for 45 minutes, and 72°C for 30 seconds and a single final elongation step at 72°C for 10 minutes. The PCR products were separated by electrophoresis on a 1.2% agarose gel, stained, and visualized on a transilluminator with UV light. Fragments of approximately 455 bp were considered positive [20].

### 2.3. Indoleacetic Acid (IAA) Production

The evaluation of IAA production by endophytic species of *I. cangae* was performed according to Bent et al. [21] with modifications. The colonies were grown in LB medium and shaken at 100 rpm and at 30°C, for 18 h. A 100 *μ*L aliquot of the LB medium with colonies was inoculated into 3 mL of King's B medium (20 g/L protease peptone; 1.5 g/L K_2_HPO_4_; 1.5 g/L MgSO_4_.7H_2_O; 20 g/L agar, pH 7.2), supplemented with L-tryptophan (0.25 g/L) as a precursor for IAA synthesis [22]. Afterward, the vial was incubated shaking (100 rpm) at 28°C in the dark, for 72 hours. Then, a 1.5 mL aliquot of cell suspension was transferred to 2 mL microtubes and centrifuged for 10 minutes at 7,000 × *g*. The auxin levels present in the supernatant were measured after mixing 100 *μ*L of Salkowski reagent (0.1125 g FeCl_3_, 10 mL H_2_O, and 15 mL H_2_SO_4_ 96%) with 100 *μ*L of supernatant in a 96-well plate. The plate was incubated for 30 minutes under regular light at room temperature. The assay was performed in triplicate.

The development of pink color indicates the production of qualitative IAA, being the intensity of the staining directly proportional to the concentration of IAA present in the medium [17]. The absorbance was read in a spectrophotometer at 530 nm. King's B medium without inoculum was used as the negative and white control. The IAA produced was determined by building up a standard curve (adjustment *R*^2^ = 0.988) with 0, 1, 3, 5, 7, 10, and 25 *μ*g/mL IAA concentrations.

### 2.4. Siderophore Production

Production of siderophores was analyzed according to Schwyn and Neilands [23]. The colonies were grown in LB broth, at 30°C and 100 rpm, for 48 hours. A 5 *μ*L aliquot of the isolates grown on LB broth was spot inoculated on CAS medium (King's B medium, supplemented with chromoazurol S + FeCl_3_ + hexadecyltrimethylammonium) and incubated at 28°C for 4 to 7 days. The results of the test for the identification of isolates producing siderophores were considered positive when clear halos were observed around the colonies. The assay was performed in triplicate.

### 2.5. Inorganic Phosphorus Solubilization Capacity

The cultures were reactivated in LB solid medium. Aliquots of 0.5 mL of bacterial suspension (10^8^ cells/mL) were individually transferred to a 250 mL Erlenmeyer flask containing NBRIP liquid culture medium [24] added with 2 g/L of FePO_4_. The culture medium and culture medium added with inoculum-free iron phosphate were established as controls. The treatments were incubated at 28°C and shaken for 10 days. After this incubation period, the cultures were centrifuged at 5,000 ×*g* for 10 min, and the supernatant was filtered on Whatman no. 42 filter paper. To quantify the soluble P, the colorimetric method of Murphy and Riley [25] was used, subtracting the soluble P contained in the control sample (culture medium with iron phosphate without inoculation). The assay was performed in triplicate. The pH of the filtered supernatant of all samples, including the controls, was also determined.

### 2.6. Organic Phosphorus Mineralization

The organic phosphorus mineralization test was performed according to Dash et al. [26], with modifications. Cultures grown in LB medium (10 g/L) were spot inoculated on Petri dishes containing selective agar sodium phytate medium (10 g/L D-glucose, 4 g/L sodium phytate, 2 g/L CaCl.2H_2_O, 5 g/L NH_4_NO_3_, 0.5 g/L KCl, 0.5 g/L MgSO_4_, 0.01 g/L MnSO_4_, 15 g/L agar, pH 7.00 at 25°C) for identification of the potential for organic phosphorus mineralization. The plates were incubated at 28°C for 15 days. The formation of a clear halo zone around the colonies during the 15 days shows phosphate mineralization. The assay was performed in triplicate.

### 2.7. Genomic DNA Isolation, PCR, and 16S rDNA Sequencing

The sequencing of the 16S rRNA gene, a preserved region used for phylogenetic studies, was carried out for the molecular identification of selected bacterial isolates from different parts of the plant.

The total genomic DNA of the isolates was extracted with the Wizard® Genomic DNA Purification Kit (Promega Corporation/Madison, Wisconsin, EUA). The purified DNA was quantified in NanoDrop and used as a template to amplify a segment of the 16S rRNA gene by PCR using the universal prokaryotic primers 27f and 1492r [27, 28]. Each reaction was performed in a volume of 50 *μ*l in a tube containing 10 *μ*l of 5X PCR buffer (Promega Corporation/Madison, Wisconsin, EUA), 2 mM MgCl_2_ (Promega Corporation/Madison, Wisconsin, EUA), 200 *μ*M of each of the four dNTPs (Promega Corporation/Madison, Wisconsin, EUA), 0.2 *μ*M of each primer (IDT), and 1.5 U of Taq DNA polymerase (Promega Corporation/Madison, Wisconsin, EUA). The program used for amplification was as follows: 95°C for 5 min, followed by 25 cycles of 95°C for 1 min, 50°C for 1 min, and 72°C for 1 min and 30 s, and one single final elongation step at 72°C for 7 min.

The fragments obtained from the PCR reactions were separated by electrophoresis on 1% agarose gel, stained, and visualized in a transilluminator with ultraviolet light. At the end, the amplified fragments were stored in a freezer at −20°C.

The sequencing reaction was performed in the ABI 3730 DNA Analyzer, by the Instituto Tecnológico Vale using the universal prokaryotic primers 27F (5′GAGTTGATCATGGCTCAG 3′), 1492R (5′GGTTACCTTGTACGACTT 3′), and 518F (5′CCAGCAGCCGCGGTAATACG 3′) 907R (5′CCGTCAATTCMTTTRAGTT 3′). The sequences obtained were quality trimmed and analyzed using the RDP (Ribosomal Database Project) Sanger pipeline online tool, and then contigs were built in the BioEdit Sequence Alignment Editor software, version 7.0.5.3. The 16S rDNA contigs were compared with the NCBI database using the BLASTN algorithm. The FASTA files were deposited in the GenBank database with the following accession numbers (Table 1): SUB12006012 (OP456096-OP456144).

### 2.8. Inoculants Application on *I. cangae* Growth: Preliminary Studies

Aiming to promote growth on *I. cangae* and evaluate the potential of some isolated bacterial inoculants, initial studies were carried out in a greenhouse. For aquatic plant cultivation was applied 1 : 10 proportion (substrate : water). In addition to the control, the bacterial colonies used in the tests were selected from different parts of the plant, totaling 6 isolates of different endophytes originating from the leaf (*Acinetobacter soli* EL5), corm (*Acinetobacter soli* WC10 and *Bacillus* sp. EC3), and root (*Bacillus* sp. NR2, *Priestia* sp. WR9, and *Bacillus* sp. ER4). Such microorganisms were selected, taking into account both nitrogen fixation potential, siderophore production, and easy growth in LB medium. The endophytic bacteria used as inoculants were activated in LB broth at a rate of 10% v/v and incubated at 28°C at 100 rpm for 18−24 h. Subsequently, the selected microorganisms grew in 79 broth for 24 h at 30°C [16]. Subsequently, the PGPR bacteria were inoculated into the plant substrate at 1% v/v.

## 3. Results

### 3.1. Identification of Endophytes Isolated from *I. cangae*

The identification of endophytes isolated from *I. cangae* in Brazil in the period 07/2018 was based on the sequencing of the 16S rDNA gene. Because of the fragments sizes and similarity values in BLASTN Programs in the period 09/2022, the classification of most sequences was built to the taxonomic genus level. The prevailing genus (until now, because NC1, EC2, and WC7 are missing) was *Bacillus* sp. (40%), followed by *Priestia* sp. (20%), *Metabacillus* sp. (6%), *Acinetobacter* sp., *Rossellomorea* sp., and *Herbaspirillum* sp. (4.1% each), *Comamonas* sp., *Rhizobium* sp., *Staphylococcus* sp., *Enterobacter* sp., *Micrococcus* sp., *Chromobacterium* sp., *Heyndrickxia* sp., and *Rhodococcus* sp. (2% each). The species was selected when the sequence had only one species per hit and presented a percentage of similarity greater than 98% or had a size greater than 1200 bp (Table 1).

### 3.2. Selection of Representative Profiles of Isolated *I. cangae* Colonies

The disinfection of the plants to remove the epiphytic population was effective, since no bacterial growth was observed in plates with noncrushed plants. A total of 109 colonies were isolated from crushed plants in the LB agar and tested for morphological characteristics, classifying them according to the portion of the Amendoim pond where plants were collected from (West, North, or East) and the part of the plant (leaf, corm, or root). The isolates were coded according to their origem east (E), west (W), and north (N) from Amendoim pond and from different *I. cangae* parts: corm (C), leave (L), and root (R).

Among the isolated colonies and after the reactivation of the frozen cultures of the selected microorganisms only 49 isolated colonies remained viable to growth in LB medium for the following tests to evaluate the potential promotion of vegetal growth.

### 3.3. Nitrogen Fixation of Endophytic Microorganisms Isolated from *I. cangae*

Most of the isolates showed to be capable of fixing N_2_. 36 isolates (75% of the total tested) formed an aerotactic band near the surface of NFb medium, selective for nitrogen-fixing strains. Only the *Chromobacterium* sp. (EL4) strain showed no growth in NFb medium and was not evaluated (Table 1).

### 3.4. Indoleacetic Acid (IAA) Production from Endophytic Microorganisms Isolated from *I. cangae*

**Figure 1:**
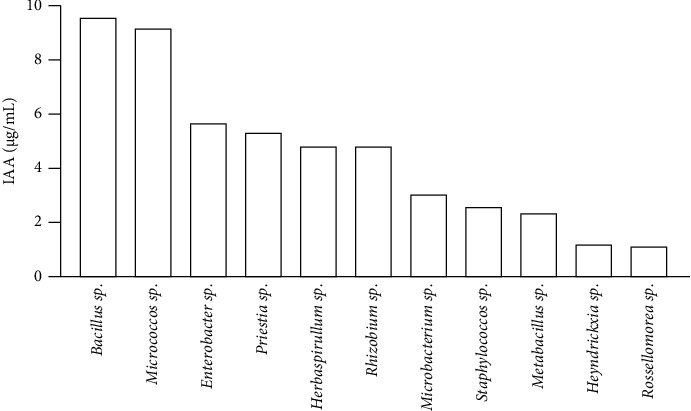
The best results from endophytic bacteria genders of *I. cangae* in IAA production (*μ*g/mL).

The production of IAA could be identified in 44 isolates.  Figure 1 shows the best results from endophytic bacteria genders in IAA production varied from 0.10 to 9.49 *μ*g/mL. 22.7% of isolated bacteria were lower than 1 *μ*g/mL, 68.2% between 1 and 5 *μ*g/mL and 9.1% higher than 5 *μ*g/mL. The isolates *Bacillus* sp. (WC6), *Microbacterium* sp. (NC2), and *Enterobacter* sp. (NL8) had the most promising results. *Rodococcus* sp. (EC4), *Acinetobacter soli* (EL5), *Acinetobacter soli* (WC10), and *Rossellomorea* sp. (WL3) were not IAA producers.

### 3.5. Production of Siderophores from Endophytic Microorganisms Isolated from *I. cangae*

Siderophores are specific binders (chelating agents) of Fe^3+^, produced by microorganisms under iron deficiency to sequester and transport this mineral into the cell metabolism [29]. Among the tested isolates, only 7 were producers of siderophores: *Priestia megaterium* (EL3), *Enterobacter* sp. (NL8), *Priestia* sp. (NL4), *Acinetobacter soli* (EL5), *Acinetobacter soli* (WC10), *Herbaspirillum aquaticum* (NC6), and *Herbaspirillum* sp. (WC9). Ten isolated bacteria (20.4%) did not grow in CAS medium, and the remainders were negative for the test (Table 1). Thereby, the colonies that show the best performance for the siderophore synthesis also are promising for ferric phosphate solubilization.

### 3.6. Phosphate Mineralization by Endophytic Microorganisms Isolated from *I. cangae*

**Figure 2:**
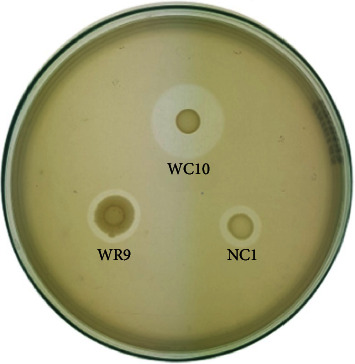
Halo zones of organic phosphate solubilization formed by three endophytes isolated—*Priestia* sp. (WR9), *Microbacterium* sp. (NC1), and *Acinetobacter soli* (WC10)—from *I. cangae* in sodium phytate agar medium.

Most of the isolates tested (78.7%) were capable of organic phosphate solubilization forming clear halo zones around the colonies in the sodium phytate agar medium (Figure 2). Isolates *Acinetobacter soli* (EL5) and *Acinetobacter soli* (WC10) showed the largest zone of solubilization. *Chromobacterium* sp. (EL4) and *Herbaspirillum* sp. (WC9) isolates lost viability and could not be evaluated.

### 3.7. Multifunctional Potential of Isolates

**Figure 3:**
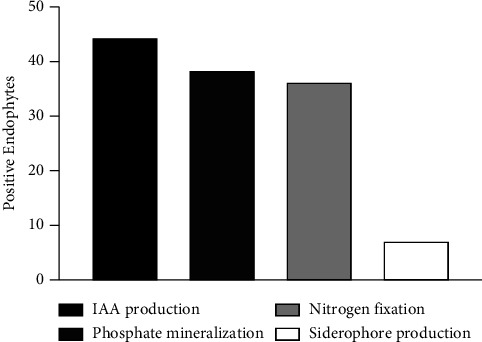
Number of positive endophytes of each test of growth promotion potential.

In total evaluated isolates, 89% were positive for the IAA production, 75% of the total tested was positive for nitrogen fixation, 78.7% were able to mineralize phosphate, and only 14.3% produced siderophores, as shown in Figure 3.

### 3.8. Inoculants Application on *I. cangae* Growth: Preliminary Studies

**Figure 4:**
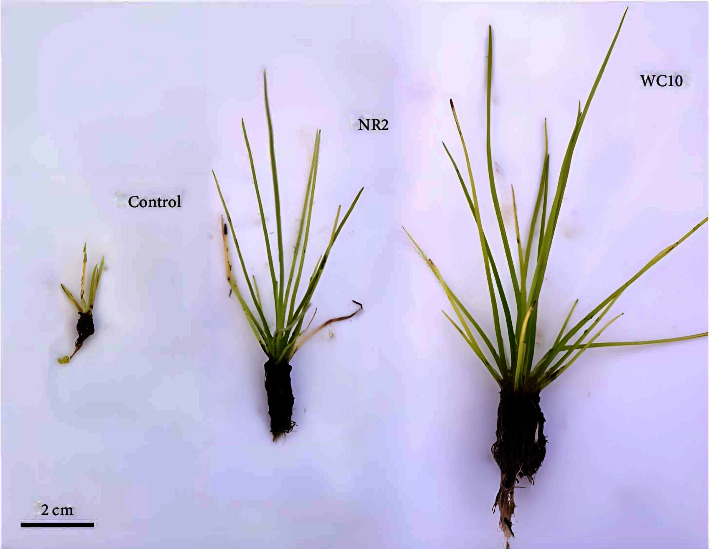
Cultivation of *I. cangae* in the absence of inoculants (control), with addition of *Acinetobacter soli* (WC10) inoculant and *Bacillus* sp. (NR2).

The inoculant application in *Isoetes cangae* enabled improvements in plant growth, such as an increase in the number of leaves, as well as a more robust/elongated leaf area and root. The treatments that used the colonies *Acinetobacter soli* WC10 followed by *Bacillus* sp. NR2 were more promising in the plant cultivation (Figure 4) promoting positive changes in the plant. The plant cultivation response variables were improved in the presence of inoculants, promoting an increase of up to 5 times the leaves number, as well as average root length in up to 4 and 4.5 times the length leaves, differing significantly (*p* < 0.05) using the test ANOVA performed with the 3 treatments.

## 4. Discussion

The assays showed that the endophytic species of *I. cangae* are diversified and promising as potential bacteria to promote plant growth through inoculation. According to Pramanic et al. [30], the endophytic microbiome increases the potential of plant-based systems, providing improvements in the ecological efficiency. The study of endophytes and their inherent functions at specific spatial and ecological locations will also aid in understanding the growth of specific plants in that location and ways of controlling their growth through endophytic microbiome modelling.

Makino et al. [31] isolated aquatic plant growth-promoting bacteria from *Lemna mino*, a water lentils, verifying that the Pelomonas sp. strain promoted IAA production, which is also observed in known land plants, while other traits, such as siderophore production and phosphate solubilization. The authors indicated that additional candidate PGPB for duckweeds could be obtained by screening bacterial isolates from diverse aquatic plants. Pramanic et al. [30] detailed the studies on microbiomes and diversity in microbial communities inhabiting the three common free-floating aquatic plants of tropical regions viz., duckweed, water hyacinth, and water lettuce, widely implicated for their bioremediation potential. Studies conducted till date reveal the prevalence and dominance of different *Bacillus*, *Rhodanobacter*, *Pseudomonas*, *Rhizobium*, *Achromobacter*, *Serratia*, *Actinobacteria*, *Proteobacteria*, *Klebsiella, and Acidobacteria*, have also been prominently reported. According to O'Brien et al. [32], duckweed-associated microorganisms were proposed to aid in a number of host-related functions, including plant defense, increased nutrient availability, phytohormones production, phytoremediation, and prevention of abiotic stress. Shehzadi et al. [33] reported that the genus *Microbacterium*, *Bacillus*, and *Halomonas* were found to be associated with wetland plants, *Typha domingensis*, *Pistia stratiotes*, and *Eichhornia crassipes*.

Castro et al. [34] isolated endophytic microorganisms from two mangrove species, *Rhizophora mangle* and *Avicennia nitida*, found in streams of two mangrove systems in Bertioga and Cananéia, SP, Brazil. The authors reported that *Bacillus* sp. was the most frequently isolated genus, comprising 42% of the species isolated from Cananéia and 28% of the species from Bertioga. Here, we emphasize the importance of the endophytic genus *Bacillus* in *I. cangae* (approximately 40%) in comparison with other bacterial genera. Ando et al. [35] also isolated a large number of *Bacillus* sp. from mangrove sediments in Japan and reported the possible ability of these isolates to degrade polluting organic compounds by fermentation. Among the isolates, the authors identified two endophytes, *B. thuringiensis* (MB4) and *B. pumilus* (MB8), which were able to control many bacterial and fungal pathogens.

Chen et al. [36] reported that endophytic bacteria were isolated from 4 species of aquatic plants: *Phragmites communis*, *Potamogeton crispus*, *Nymphaea tetragona*, and *Najas marina*. The isolated bacteria were classified into 12 genera in the *Gammaproteobacteria*, Bacilli, *Alphaproteobacteria*, *Flavobacteria,* and *Actinobacteria*. In addition, different strains were isolated from different parts of the 4 plants, suggesting the different parts of the 4 plants harbored different endophytic bacteria, similar to this work that isolated bacteria from leaf, root, and corm of *I. cangae*. Chen et al. [36] found that endophytic bacteria *Pseudomonas* sp., *Enterobacter* sp., *Aeromonas* sp., *Flavobacterium* sp., *Klebsiella* sp., *Pantoea* sp., and *Paenibacillus* sp. have a P-solubilization capacity and could be used as inoculants and promoters to increase P-uptake by plants, as suggested in this work by *Enterobacter* genus. Pontes et al. [37] isolated bacteria from *Hordeum vulgare* L. with potential to be used as inoculant in the same species, producing indolic compounds and siderophores, as well as phosphate solubilization.

The results of the nitrogen fixation capacity assays indicate that endophytic species have an important role in nitrogen fixation as well, covering 75% of the isolated species, which favors the growth and physiology of the plant. Moreira and Siqueira [38] highlighted the great relevance of nitrogen-fixing microorganisms not only in the plant rhizosphere but also in the soil and philosphere. In the aquatic environment, where *I. cangae* lives, nitrogen fixation supports primary productivity [39, 40] and plays a critical role in maintaining the balance of the nitrogen pool in combination with denitrification [41, 42].

In this study, the *nifH* gene was found in 20.4% of the colonies. Other genes are also important for the molecular indication of this feature. For example, in *K. pneumoniae*, 20 unidentified genes are grouped together in a very compact organization in a chromosomal region encompassing 24,206 base pairs organized in 8 operons: *nifJ*, *nifHDKTY*, *nifENX*, *nifUSVWZ*, *nifM*, *nifF*, *nifLA*, and *nifBQ* [43]. In addition, Kuklinsky-Sobral et al. [44] and Zehr et al. [45] reported that the *nifH* gene could not be amplified from some symbiotic bacteria, which may be due to variability of the *nifH* gene sequence.

The IAA tests were very satisfactory regarding the potential of isolated colonies in relation to plant health, since this substance affects the root morphology, increasing the length and root hair number (Figure 3). Among auxins, IAA is the most studied and produced by bacteria [46], with practical application in plant growth promotion [47]. There is also increasing evidence that IAA is a signaling molecule in microorganisms that can act as a reciprocal signaling molecule in microbe plant interactions, as it affects gene expression in some microorganisms [48]. Leite et al. [49] also evaluated that 78% of the isolates from cassava produced IAA and 31% of the isolates were able to solubilize inorganic phosphate. The identification of 19 isolates allowed the grouping into six bacterial genera, namely: *Achromobacter*, *Bacillus*, *Burkholderia*, *Enterobacter*, *Pantoea*, and *Pseudomonas*. It should be noted that the genera *Bacillus* sp. and *Enterobacter* sp. were reported and sequenced in the present work, and 78.7% of the isolates were able to mineralize organic phosphate as indicated above.

The endophytic bacterial strains were important for siderophore production as well, improving the Fe availability to the associated plant. Pontes et al. [37] highlighted that endophytic bacterial strains that produce siderophores are important for the synthesis of the bacterial consortium, since they can reduce phytopathogens proliferation by iron chelation. In addition, these compounds can reduce the availability of metals in the rhizosphere, reducing their toxicity to plants and their own microorganisms [50]. This finding is important to understand the ecology of the plant that lives in an iron-rich region such as Canga, in Carajás. In this sense, the isolated bacteria showed great ecological importance and adaptation to impacted environments, in addition to the possibility of being used in bioremediation techniques. Dimkpa et al. [51] suggest that siderophore-producing and auxin-producing bacteria simultaneously can be potential candidates for microbe-assisted phytoremediation of metal contamination; they found representative *Streptomyces* sp. species producer of three hydroxamate siderophores that promote auxin synthesis in the presence of some ions by chelating these metals and making them unavailable for uptake by the plant and inhibiting the synthesis of auxins.

The assays for organic phosphate mineralization by the isolated colonies were promising, confirming that about 50% of bacterial isolates were capable of sodium phytate mineralization. A reduced availability of phosphorus limits plant growth, being essential for its metabolism [52–54]. Thus, it is important for the health of the plant to contain endophytic microorganisms that have this ability. Mineralization is measured by phosphatase enzymes produced by plant and microbe in the soil, catalyzing organic phosphate hydrolysis [55] and releasing inorganic phosphorus for plant uptake [56]. The results were similar to those reported by Junior and Oliveira [57], who observed that 33.2% of rhizobium isolates collected in Amazonian soil produced a hydrolysis halo zone, while Pedrinho et al. [58] obtained 46.55% of solubilizing bacterial isolates. Fernández et al. [59] highlighted that the diversity of microorganisms capable of solubilizing inorganic phosphate varies across each type of soil and environment. The same authors verified in their study that only 0.06% of the total bacteria had the potential to solubilize inorganic phosphate.

When bioprospecting for endophytic bacteria in *I. cangae*, the genus *Bacillus* was the most observed, being a complex group for species differentiation. Besides being cosmopolitans, they are related to biogeochemical cycles, such as carbon and nitrogen. Several studies confirmed the potential of Bacilli as biocontrol agents and encourage their use in agriculture [60]. They are capable of synthesizing antimicrobial substances such as ethylparaben, lipopeptides [61], and enzymes that attack the phytopathogenic fungi [62].

Multiple phylogenetic studies and comparative genomic analyses have been conducted to clarify the taxonomy of the *Bacillus* genus, according to Gupta et al. [63]. Thus, recent research indicates that *Heyndrickxia* sp., *Priestia* sp., and *Rossellomorea* sp. are products of the genus *Bacillus* sp., corresponding to 4%, 20%, and 4% of the genera described in this study (Table 1), respectively.

Nutaratat et al. [64] identified the genus *Enterobacter* sp. DMKU-RP206 in a semiaquatic plant, a rice phyllosphere bacterium that possesses plant growth-promoting traits. The bacterium was assessed on plant growth-promoting traits including indole-3-acetic acid (IAA) production. Phosphate solubilization, ammonia production, and antagonism to fungal plant pathogens, as well as siderophore production, were shown by this bacterium. In this work, the genus *Enterobacter* was also identified and was effective in the IAA production.

*Acinetobacter soli* was characterized in this work in two bacterial isolates that presented expressive results in the assays. This species has huge metabolic and nutritional versatility and a high degree of hostile and diverse environmental adaptation ability. It uses different carbon sources and can grow in diverse temperature and pH conditions, it is disinfectant-resistant, tolerates low humidity rates, can adhere to and form biofilms in soil grains, which contribute to its persistence in different environments [65–68]. Corroborating our observations, studies carried out by Kleingesinds [69] demonstrate that *Acinetobacter soli* can potentially be used as an inoculant in *Sacrum* sp., as well as its ability for nitrogen fixation and IAA production.

Silva et al. [70] analyzed a high number of colonies isolated from *Aloe Vera* belonging to the phyla Proteobacteria and Firmicutes, followed by *Actinobacteria* and *Bacteroides*. Their results were similar to those found in this study, in which the genera *Acinetobacter* sp. and *Bacillus* sp. were identified as members of the phyla Proteobacteria and Firmicutes, respectively. These genera have previously been reported as growth promoters, with the potential to be used in phytoremediation [71, 72], biostimulation, biocontrol, and biofertilization [73, 74].

According to Anudechakul et al. [75], *Acinetobacter* sp., isolated from the roots of Pontederia, was found to facilitate and enhance the removal of chlorpyrifos by the aquatic plant. Shiomi et al. [74] performed bioprospecting to discover endophytic bacteria for biological control of coffee leaf rust and identified the genus *Acinetobacter* sp., as well as species of the genus *Bacillus* sp., among other endophytic species. The authors highlighted the fact that the endophytic bacteria isolates showed activity when applied before the pathogen, suggesting that these isolates may act by antibiosis, lysis of pathogen, competition, or induction of systemic resistance in the host. Ishizawa et al. [76] reported that *Acinetobacter calcoaceticus* P23 and *Pseudomonas fulva* Ps6 increased growth and biomass production when inoculated in hydroculture systems. Yamaga et al. [77, 78] reported that the PGPB for duckweeds, *Acinetobacter calcoaceticus P23*, which increases the number of duckweed fronds (leaf-like structures), was isolated from *Lemna* sp. The genus *Acinetobacter* sp. was also evidenced in this work, indicating its promise for promoting plant growth due to its properties to establish, produce IAA, and mineralize phosphate.

*Herbaspirillum* sp., also identified in this study, can usually fix atmospheric N_2_ in microaerobic conditions and grow in the presence of N_2_ as only nitrogen source. It is mainly associated with grasses, endophytically colonizing roots, stems, and leaves. However, according to Dobritsa et al. [79], *Herbaspirillum aquaticum* (IEH 4430, ATCC BAA-1628, DSM 21191) cannot fix N_2_ and neither grows in JNFb medium, which is mainly used to isolate N_2_-fixing bacteria, such as *Azospirillum* sp. and *Herbaspirillum* sp. [80]. The study corroborates our result, not confirming N fixation by this species, as highlighted in Table 1. According to Stoltzfus et al. [81], the *nifH* and *nifD* genes were not identified by the PCR technique with the primers in preconized conditions and neither the nitrate reductase enzyme was observed.

The genus *Rhizobium*, which is traditionally considered as legume endosymbionts and has generally been isolated from nodules [82], was also identified in this work. However, large populations of rhizobia are found in the soil and rhizospheres and are defined as endophytic species. Rhizobia have great environmental importance, since they induce root nodules and fix atmospheric N_2_ in most legume species in exchange for carbon [83]. As in the present study, Lin et al. [82] identified a *Rhizobium straminoryzae* strain, investigating the bacterial diversity from rice straw in Taiwan. The researchers analyzed the presence of *nifH*, but the gene was not detected with the primers FGPH19/PolR and AQER/PolF. The results of nitrogen fixation for this species were not positive, as seen in Table 1. However, the performance of this species regarding the mineralization of phosphate, siderophore production, as well as IAA production was relevant.

According to Pramanic et al. [30], there are many studies on the taxonomical and functional aspects of microorganisms associated with terrestrial plants. However, the microbiome of aquatic plants is not much explored. In future trials the endophytic microorganisms can be used to control diseases caused by bacteria, fungi, and nematodes, since they compete for colonization of the similar niches occupied by these pathogens and stimulate the activation of plant defense response, leading to an increase in resistance [84]. The endophytic bacteria can act in synergism, favoring the growth and development of *I. cangae* and improving the health and performance of plants.

The preliminary study using the bacterial inoculants was very promising. The selected bacteria that have the ability to make the phosphorus present in the soil soluble and make it available to the plant, among other properties already described in this report, promoted up to 5-fold improvements in *I. cangae* growth. Future steps consist of optimizing the inoculant consortium through different implementation techniques in the culture substrate, such as cell immobilization or centrifugation of the cultivated bacterial species. When inoculants are applied in consortium, the antibiosis exerted by inoculant bacteria on pathogens acts through different mechanisms, such as synthesis of antimicrobial substances, competition for space and nutrients, secretion of lytic enzymes, change in pH, and/or synthesis of volatile compounds, representing losses in the cultivation of such species [5, 33, 55, 61, 62, 85, 86].

Thus, in the present study, techniques were applied to identify bacterial isolates with an important role in the construction of an inoculant consortium to be implemented in the *I. cangae* preservation, according to Chai et al. [87] and Olanrewaju and Babalola [88]. In addition, the activities presented by the bacterial isolates deserve to be investigated from the perspective of biotechnology resources.

## 5. Conclusions

The cultivation and isolation of microorganisms present in the leaf, root, and corm of the *I. cangae* species, from different collection points, allowed the identification of several endophytic bacterial isolates that were evaluated in relation to their potential application as plant growth-promoting microorganisms. In the present study, 49 bacterial cultures were isolated and identified. Among them, several showed potential for biological nitrogen fixation (35), detection of the *nifH* gene (10), siderophore producers (7), phosphate mineralizers (37), and auxin producers (44). This study represents the first effort to investigate a tropical endemic and ancestry plant associated with rare and oligotrophic environments, attesting to the well-known relevance of endophytic bacteria biodiversity. These endophytic bacteria represent, as in other groups, a very important technology to increase plant production, potentiating the conservation efforts of *I. cangae*.

## Figures and Tables

**Table 1:** Molecular identification with similarity value in BLASTN programs in the period 09/2022, GenBank database with the isolates accession numbers, and microbiological quantification tests for N fixation-capacity, P mineralization, siderophores, IAA production, and *nifH* presence of endophytes isolated from *I. cangae* in Brazil in the period 07/2018.

Strain	Accession	Organism	Similarity value (%)	Nitrogen fixation	Siderophore production	Phosphorus mineralization	*NifH* gene	IAA production
EC1	OP456132	*Bacillus* sp.	99.48	+	NG	+	+	+
EC2	OP456126	*Metabacillus* sp.	98.47	−	−	−	−	+
EC3	OP456140	*Bacillus* sp.	99.78	+	−	+	−	+
EC4	OP456116	*Rhodococcus* spp.	97.05	I	NG	−	−	−
EL1	OP456109	*Comamonas* spp.	99.72	+	−	−	−	+
EL3	OP456123	*Priestia megaterium*	99.73	+	+	+	−	+
EL4	OP456115	*Chromobacterium* sp.	99.02	NV	NV	NV	−	−
EL5	OP456128	*Acinetobacter soli*	98.73	+	+	+	+	−
ER1	OP456108	*Priestia* sp.	98.59	+	−	+	−	+
ER2	OP456119	*Priestia* sp.	98.41	I	NG	+	−	+
ER3	OP456142	*Bacillus* sp.	99.85	+	−	+	+	+
ER4	OP456139	*Bacillus* sp.	99.17	+	−	−	−	+
NC1	OP456125	*Microbacterium* sp.	98.09	+	−	+	−	+
NC2	OP456114	*Micrococcus* sp.	97.48	I	−	−	−	+
NC3	OP456144	*Bacillus* sp.	98.72	+	−	+	−	+
NC4	OP456103	*Priestia* sp.	100.00	+	−	+	−	+
NC5	OP456121	*Priestia megaterium*	99.32	−	−	+	−	+
NC6	OP456122	*Herbaspirillum aquaticum*	99.17	I	+	+	−	+
NC7	OP456098	*Heyndrickxia* sp.	98.68	+	−	+	−	+
NL1	OP456143	*Priestia megaterium*	98.54	+	NG	+	+	+
NL4	OP456120	*Priestia* sp.	98.38	I	NG	+	−	+
NL5	OP456135	*Rossellomorea marisflavi*	99.26	+	NG	+	+	+
NL6	OP456099	*Priestia koreensis*	98.70	+	−	+	−	+
NL7	OP456113	*Priestia* sp.	97.28	I	−	+	−	+
NL8	OP456110	*Enterobacter* sp.	98.94	+	+	+	−	+
NR1	OP456136	*Bacillus* sp.	100.00	+	NG	+	−	+
NR2	OP456130	*Bacillus* sp.	97.99	+	−	−	+	+
NR3	OP456137	*Bacillus* sp.	99.83	+	NG	+	−	+
NR4	OP456138	*Bacillus* sp.	100.00	+	NG	+	−	+
NR5	OP456101	*Bacillus* sp.	99.72	+	−	+	−	+
NR6	OP456102	*Bacillus* sp.	99.14	+	−	+	−	+
NR8	OP456118	*Priestia* sp.	98.05	I	−	+	−	+
WC1	OP456107	*Metabacillus* sp.	97.62	+	−	−	−	+
WC2	OP456112	*Bacillus* sp.	97.05	+	−	+	−	+
WC3	OP456131	*Bacillus* sp.	99.43	+	−	+	−	+
WC4	OP456133	*Bacillus* sp.	99.49	+	NG	+	+	+
WC5	OP456106	*Staphylococcus* sp.	98.55	+	I	+	+	+
WC6	OP456105	*Bacillus* sp.	97.29	+	−	−	−	+
WC7	OP456127	*Metabacillus* sp.	98.32	−	−	−	−	+
WC8	OP456097	*Metabacillus* sp.	97.32	+	I	−	−	+
WC9	OP456124	*Herbaspirillum* sp.	97.32	+	+	NG	−	+
WC10	OP456129	*Acinetobacter soli*	98.24	+	+	+	+	−
WL3	OP456117	*Rossellomorea* sp.	99.12	I	−	+	−	−
WL4	OP456134	*Rhizobium straminoryzae*	98.56	−	+	+	−	+
WR10	OP456096	*Bacillus* sp.	98.97	+	−	+	+	+
WR2	OP456100	*Bacillus* sp.	98.71	I	+	+	−	+
WR4	OP456104	*Bacillus* sp.	99.29	+	−	+	−	+
WR5	OP456141	*Bacillus* sp.	99.47	+	−	+	−	+
WR9	OP456111	*Priestia* sp.	99.29	+	−	+	−	+

NG: no growth in culture medium; NV: no viability; I: indeterminate. The isolates were coded according to their origem from the Amendoim pond as E (east), W (west), and N (north) and from different *I. cangae* parts: corm (C), leaf (L), and root (R).

## Data Availability

The data presented in this study are available in the article.
